# Eyeing the Extracellular Matrix in Vascular Development and Microvascular Diseases and Bridging the Divide between Vascular Mechanics and Function

**DOI:** 10.3390/ijms21103487

**Published:** 2020-05-15

**Authors:** Brahim Chaqour, Charles Karrasch

**Affiliations:** 1 Department of Cell Biology, SUNY Downstate Medical Center, Brooklyn, NY 11203, USA; ckarrasck@downstate.edu; 2 Department of Ophthalmology, SUNY Downstate Medical Center, Brooklyn, NY 11203, USA

**Keywords:** retina, angiogenesis, extracellular matrix, growth factor, ischemia, ischemic retinopathy, diabetic retinopathy, neovascularization, CCN2, CTGF, basement membrane, stiffness

## Abstract

The extracellular matrix (ECM) is critical in all aspects of vascular development and health: supporting cell anchorage, providing structure, organization and mechanical stability, and serving as a sink for growth factors and sustained survival signals. Abnormal changes in ECM protein expression, organization, and/or properties, and the ensuing changes in vascular compliance affect vasodilator responses, microvascular pressure transmission, and collateral perfusion. The changes in microvascular compliance are independent factors initiating, driving, and/or exacerbating a plethora of microvascular diseases of the eye including diabetic retinopathy (DR) and vitreoretinopathy, retinopathy of prematurity (ROP), wet age-related macular degeneration (AMD), and neovascular glaucoma. Congruently, one of the major challenges with most vascular regenerative therapies utilizing localized growth factor, endothelial progenitor, or genetically engineered cell delivery, is the regeneration of blood vessels with physiological compliance properties. Interestingly, vascular cells sense physical forces, including the stiffness of their ECM, through mechanosensitive integrins, their associated proteins and the actomyosin cytoskeleton, which generates biochemical signals that culminate in a rapid expression of matricellular proteins such as cellular communication network 1 (CCN1) and CCN2 (aka connective tissue growth factor or CTGF). Loss or gain of function of these proteins alters genetic programs of cell growth, ECM biosynthesis, and intercellular signaling, that culminate in changes in cell behavior, polarization, and barrier function. In particular, the function of the matricellular protein CCN2/CTGF is critical during retinal vessel development and regeneration wherein new blood vessels form and invest a preformed avascular neural retina following putative gradients of matrix stiffness. These observations underscore the need for further in-depth characterization of the ECM-derived cues that dictate structural and functional properties of the microvasculature, along with the development of new therapeutic strategies addressing the ECM-dependent regulation of pathophysiological stiffening of blood vessels in ischemic retinopathies.

## 1. Introduction

**Figure 1:**
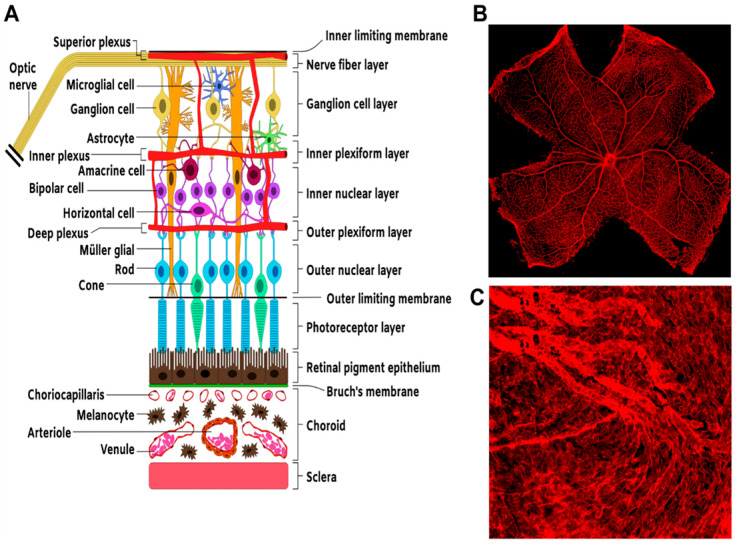
Structure and organization of the neural and vascular retina. (**A**) Schematic. Representation of section of the retina showing the overall arrangement of retinal neural layers and the basic vascular circuitry. (**B**) Flat mount preparation of a mouse retina showing IB-4-stained retinal vasculature. (**C**) Flat mount preparation of IB-4-stained choroidal vasculature.

The hallmark of many forms of blinding diseases is a disrupted oxygen supply to the neural retina and subsequent loss of function of photosensitive neurons required for photo-transduction and transmission of visual information from the retina to visual processing and cognitive centers in the brain [1,2]. Oxygen and nutrient supply to the retina is derived from two separate and remarkably different vascular beds supplying the inner and outer parts of the retina: the retinal vasculature, a sparse but hierarchically specified circulation; and the choroid, a dense and more permeable vasculature with little arteriovenous specification, respectively (Figure 1). These vascular beds often sustain injurious alterations associated with diabetes, trauma, hyperoxia, dyslipidemia, or the interactions of genetic predisposition, environmental insults, and ageing [3].

The retina’s high metabolic and oxygen demands make it highly susceptible to these injurious stimuli, which cause an arrest of vascular development, endothelial dysfunction, vaso-obliteration and/or vascular occlusion [4,5,6,7]. The subsequent vascular pathological response, especially in intraocular vascular diseases, generates disorganized, hyperpermeable, and/or tortuous capillaries that leak into the interface between the vitreous and the retinal tissue, attracting fibroglial elements and causing severe hemorrhage, retinal detachment, and vision loss [8,9]. These are the clinical sequalae of neovascular and fibrovascular diseases of the eye such as retinopathy of prematurity (ROP), proliferative diabetic retinopathy (DR), and/or proliferative vitreoretinopathy (PVR). Wet age-related macular degeneration (AMD), which causes blindness in elderly populations, is characterized by the sprouting of new vessels from the choriocapillaris through Bruch’s membrane into the sub-retinal space or the retina layers [10]. Diabetes-related abnormalities of the vitreoretinal interface may promote the development of diabetic macular edema (DME), the most common cause of visual loss in DR patients [11,12]. In DME, the macula and the disk may adhere firmly to the posterior hyaloid, contributing further to blood–retinal barrier breakdown and traction on the macula [13].

Although retinal vasculopathies are multifactorial progressive diseases, endothelial dysfunction appears to play a key role in their pathogenesis and pathophysiological mechanisms. Indeed, the endothelium has a limited self-repair capacity, being made of terminally differentiated cells with low regenerative potential [14]. Chronic exposure to hyperglycemia as in non-proliferative DR or hyperoxia as in stage I ROP initiates an array of negative intracellular events such as increased oxidative stress (i.e., imbalance between production and scavenging of reactive oxygen species) and dysregulated repair processes [15,16]. Subsequently, vascular cells undergo apoptosis as a result of increased membrane lipid peroxidation and oxidative alterations of macromolecules essential for cellular functions. The ensuing discordance between vascular supply and tissue demand for oxygen and nutrients produces severe hypoxia and the mounting of a hypoxic response that causes detrimental preretinal and intravitreal neovascularization. This secondary angiogenesis is well characterized as a fundamental pathological feature of proliferative DR and stage II ROP, because it does not allow a normal revascularization of the ischemic retina despite a strong hypoxic response that culminates in enhanced production of proangiogenic factors [17,18]. For unknown reasons, ischemic retinas seem to be unfavorable to a reparative, developmental-type angiogenesis [19,20]. Anti-vascular endothelial growth factor (VEGF) therapy commonly used to stem neovascularization remains ineffective in numerous patients with these ischemic retinopathies [21,22]. In addition, anti-VEGF drugs may compromise the in vivo requirement for VEGF in neuroprotection and synaptic function [8,23]. Notwithstanding some benefits of anti-angiogenic therapies [21,24], anti-VEGF therapy does not benefit all patients with ischemic retinopathy indicating that, such treatments do not affect all mechanisms of microvascular dysfunction.

Matrix stiffness, one of the sources of mechanical stimulation, often precedes microvascular diseases and drives their progression by altering cellular behaviors [25,26]. Matrix stiffness depends on the composition, density, chemistry, and architecture of major globular and fibrillar proteins [27,28]. Matrix stiffness is well recognized as an independent regulator of cell migration, proliferation, stem cell differentiation and cancer cell cycle entry [29,30]. The relative proportions and physical and chemical properties of the large ECM proteins such as collagens, elastin and glycoproteins regulate the deformability of large veins and arteries, and stiffening of these large blood vessels has been associated with well-known qualitative and quantitative changes of the matrisome components [31,32]. Compliance of small caliber vessels like retinal and brain capillaries and precapillary venules depends more on the properties and their basement membrane (BM) and the subendothelial matrix, which is enriched in matricellular proteins like cellular communication network 1 (CCN1) and CCN2 (aka connective tissue growth factor) [27,33,34].

The scientific literature on the ECM and central nervous system (CNS), including the retina, is vast, and it is therefore impossible to cover this topic completely in a single article. Herein, we sought to provide an overview and specific examples of ECM functions in retinal vascular development and pathologies. We considered the importance of the ECM as both a scaffold within which retinal cells are built and as a determining factor of retinal vascular health and disease. Emphasis is placed on the regulation and function of CCN2/CTGF, a major component of the vascular matrix of the retinal and brain microvasculature. The interested reader is also referred to review articles describing other aspects of the ocular ECM and its relevance in different biological and clinical settings [35,36,37].

## 2. Blood Vessel Development and Organization in the Retina

In rodents, development of the retinal vasculature involves emergence of endothelial tubes from the optic nerves, their ensheathment with mural cells, radial growth of the vascular tubes at the retinal-vitreal interface (postnatal day (P)1-P9), and sprouting into the outer plexiform layer (ONL) and the inner plexiform layer (INL) to form the deep (P7–P11) and intermediate capillary plexuses (P11–P17), respectively. This angiogenic process occurs without perturbing the existing retinal architecture due to exquisitely well-orchestrated cross-talks among neural cells, glial cells, and the invading blood vessels [38,39]. Retinal ganglion cells (RGCs), the principal respondents to hypoxic stimulus, regulate the release of a number of major angiogenic factors capable of reinstating vascular supply to an ischemic retina. As they mature into functional neurons, these cells become avid consumers of oxygen and nutrients and spontaneously drive blood vessel formation as they sense the local environmental hypoxia [40]. The molecular effectors of the hypoxic stimulus in neuronal cells include, but are not limited to, adenosine, fibroblast growth factor (FGF), and succinate, as well as mechanical factors associated with vasodilation and increased blood flow [39,41,42,43,44]. The interaction of neuronal cells with ECM proteins is itself critical for their own survival and potential regeneration. The trajectory of RGC axons along the inner limiting membrane (ILM) keeps them in direct contact with the BM as they exit the retina. The laminins and dystroglycan, a laminin-binding transmembrane protein are critical for neuronal cell survival, growth, and possibly regeneration [45].

Meanwhile, the Müller glial cells, the radial astroglia of the retina, similarly exhibit unique properties that are critical for retinal vessel formation, regeneration, and response to injury [46,47]. Müller cells span all of the retinal layers, from the vitreal surface to the subretinal space. They ensheath the retinal neurons, cushioning them against mechanical stress but also bridging them with the vasculature. Müller cells secrete pigment epithelium-derived factor (PEDF), thrombospondin-1, and glial cell line-derived neurotrophic factor, all of which fortify the vascular endothelial barrier [48]. Müller cell loss in transgenic mice resulted in several vascular defects, including telangiectasias, loss of blood–retinal barrier integrity, and intraretinal neovascularization [49]. In the ischemic brain, astrocytes provide an antiproliferative environment for brain endothelial cells (ECs). Müller cells may likewise play the same role for retinal ECs. Such an angiostatic environment stirs blood vessel growth toward the vitreous and away from the ischemic neural retina under ischemic conditions. Müller cells are also a major source of ECM proteins in the retina. As shown in our recent work, the Müller cell secretome includes the matricellular protein CCN2/CTGF, which plays a major role in vessel development and barrier integrity, although the precise function of Müller cell-derived CCN2/CTGF remains to be determined [50].

## 3. The Vascular Matrix in the Retina

**Figure 2:**
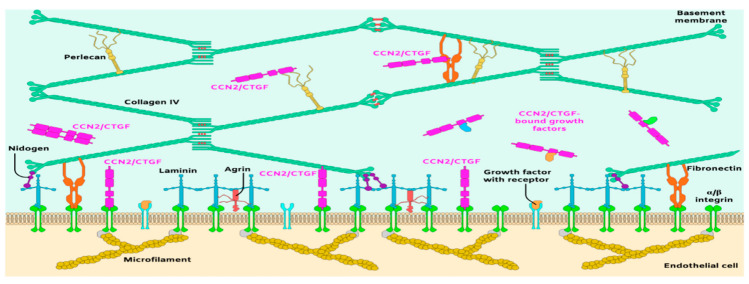
Schematic representation of the principal molecular components of the subendothelial matrix and basement membrane of blood vessels.

A basic model for framing the important but complex roles of the ECM in blood vessel formation and function is the anchorage of vascular cells on a stabilizing vascular BM, a multilayered structure that provides critical support and promotes cell adhesion, migration, and survival [35,51]. The vascular BM has a thickness of 20–200 nm and consists of a three-dimensional network predominantly composed of at least 20 ECM proteins [52]. The organization and scaffolding of four major glycoprotein families, laminins, collagen IV isoforms, nidogens, and heparan sulfate proteoglycans (HSPG) in the BM is required for proper capillary morphogenesis, vascular wall integrity and homeostasis (Figure 2). Consequently, any change in the proportion of these molecules is associated with systematic disruption of angiogenesis and/or breach of the vascular barrier, as seen in acute and chronic neuropathological settings [37]. Type IV collagen molecules, which consist of two α_1_ and one α_2_ subunits (2α_1_, 1α_2_(IV)), self-assemble to form a branching network that include NC1 and 7S domains with lateral associations. Knockout of the type IV collagen α_1_ or α_2_ subunit gene did not prevent BM formation and assembly, but reduced its stability and caused mouse early embryonic lethality [53]. Reduction in type IV collagen levels destabilized the capillary wall and caused porencephaly and hemorrhagic stroke [54], indicating that type IV collagen is critical for the integrity of the CNS microvascular wall. Similarly, laminins, the most abundant non-collagenous BM proteins, are critical for vascular morphogenesis and integrity. They consist of five α chains, four β chains, and three γ chains that combine to form 16 laminin isoforms, of which laminin 111, 211, 411, and 511 are found in the vasculature [55]. The loss of function of α_2_, 411 or 511 weakened the vascular barrier causing excessive hemorrhages in the CNS and embryonic lethality [52,56].

The laminin–collagen IV networks are linked to nidogen and HSPGs. Nidogen-1 and -2 (aka entactin-1 and -2) are important for connecting and stabilizing the self-assembled layers of laminin and collagen IV [57]. In the retina, both nidogen-1 and -2 are present in vascular and Bruch’s membranes [58]. Loss of function of nidogen-1 and -2 in mice resulted in lethality shortly before or at birth [59], suggesting that the interaction of nidogen with type IV collagen and laminin stabilizes the BM under mechanical stress. The vascular BM is similarly stabilized by HSPGs including perlecan, agrin and collagen XVII [51]. Some of these have high affinity to and tether growth factors such as FGF, transforming growth factor beta (TGF-β), glial-derived neurotrophic factor, and VEGF [60]. These ECM embedded factors are released to interact with their own receptors and mediate, at least in part, cell growth and behavior.

There are other small proteins that are associated with the BM or subendothelial matrix and provide additional cell–matrix interaction points and receptor binding activities [61,62,63]. Expression of some of these proteins depends on the developmental and physiological state. They include fibulin-1 and -2, thrombospondin-1, secreted protein acidic and rich in cysteine (SPARC), tenascins and the CCN family of proteins [64,65,66]. Many of these proteins function to regulate collagen or elastin fibril formation and size or serve as ligands for cell surface adhesion receptors. Deletions and/or mutations of most of these proteins (e.g., thrombospondins, tenascins, or SPARC) cause only mild or no vascular defects suggesting that their role in vascular structure and function is either negligible or easily compensated for by other proteins [67,68]. Loss of their function appeared to weaken tissue response to ischemia or diabetes suggesting a potentially important function of these proteins in tissue remodeling and repair [69,70]. However, our understanding of the complexity of the vascular matrix continued to grow with the characterization of the CCN proteins: a new family of six members, two of which, CCN1/CYR61 and CCN2/CTGF are critical for vascular development and organ functions [27,71,72]. These proteins associate with the ECM and cell surface but do not assume a structural role [73]. Instead, they act temporally and spatially to provide signals that modify cellular responses or fine tune growth factor activity during vascular development and microvascular diseases. Loss of function of these proteins cause severe vascular defects and embryonic or perinatal lethality, setting them apart from other matricellular proteins of the vascular matrisome [74,75]. In the following sections, we will focus on the intriguing and somewhat controversial biological functions of the CCN2/CTGF and their implications in vascular development and microvascular diseases of the eye.

## 4. CCN2/CTGF Structure and Function

**Figure 3:**
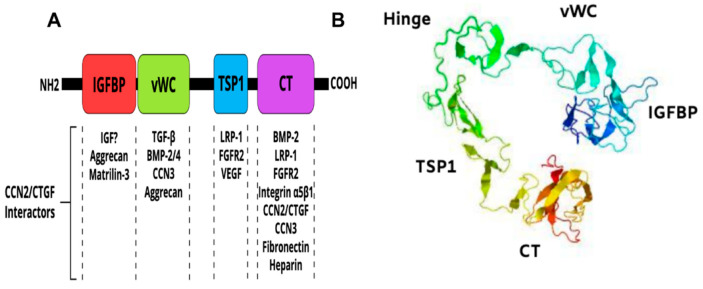
Multimodular structure of the CCN2/CTGF protein. (**A**) Schematic diagram of the constitutive domains of the CCN2/CTGF protein. Potential CCN2/CTGF interactors are indicated. (**B**) Predicted three-dimensional model of the CCN2/CTGF protein with the highest confidence score given by I-TASSER software. The protein seems to have a globular appearance with a wide “U”-shaped arrangement of its domains.

CCN2/CTGF is a member of the CCN family of matricellular proteins, which consists of six structurally conserved cysteine-rich proteins with different tissue distributions and functions [72,76]. CCN2/CTGF is a 38-41-kDa protein composed of an N terminal secretory peptide followed by four conserved domains with sequence homologies to insulin-like growth factor binding protein (IGFBP), von Willebrand factor type C repeat (vWC), thrombospondin type I repeat (TSP1), and a carboxyl-terminal (CT) domain that contains a cysteine-knot motif (Figure 3A). The primary sequence includes a series of 38 cysteine residues that are strictly conserved in their position and number among five members of the CCN family. A 3D model of the CCN2/CTGF protein as determined by I-TASSER software [77] is shown in Figure 3B. The four domains of the protein fold into a three-dimensional structure dictated by the amino acid composition, succession and the different electrostatic charges on their surfaces.

CCN2/CTGF is the most studied of the CCN family proteins by virtue of its widespread expression pattern and importance in fibrovascular diseases and cancer. CCN2/CTGF has tightly regulated expression patterns and exhibit ECM-like structural features and growth factor-like activities including modulation of cell motility, adhesion, proliferation, survival, ECM protein expression and reprogramming of gene expression [34,78,79,80]. As such, this molecule plays a critical role in blood vessel formation and regeneration [27,34,50,81].

## 5. CCN2/CTGF Expression during Embryonic and Fetal Tissue Development

The CCN2/CTGF gene, which is located within chromosome 6 q22.1-23.2 is highly conserved in vertebrate species. Comprehensive meta-analysis identified several functional single nucleotide polymorphisms (SNPs) in the non-coding regions of the CCN2/CTGF gene increasing risk of abdominal aortic aneurysm [82], systemic sclerosis [83], nephropathy in type I diabetes [84], calcified aortic stenosis [85], and even human longevity [86]. Although the direct biological relevance of SNPs and genetic variants to disease states are viewed as suggestive, these associations reveal a potential role of CCN2/CTGF in vascular development and diseases.

The CCN2/CTGF gene was found to be dynamically expressed during murine embryonic development, most prominently in embryonic tissues that give rise to the cardiovascular, musculoskeletal, and nervous systems [87]. CCN2/CTGF was detected as early as embryonic day (E) 7.5 in the parietal endoderm, the notochord, cephalic mesenchymal cells, the first bronchial pouch, and in the roof and floor plate of the neural tube. Four upstream enhancer regions have been shown to drive tissue specific CCN2/CTGF expression, one of which is specific to vascular tissue [88]. At E.8, CCN2/CTGF appeared in the cardiovascular system including the heart myocardium, the common cardinal vein, branchial arch arteries, and the dorsal aorta, and in the ductus arteriosus thereafter. The expression of CCN2/CTGF in the first embryonic vasculature suggests a potentially important role of this molecule in endothelial progenitor cell differentiation and proliferation.

Likewise, CCN2/CTGF expression fluctuates during human embryonic development. CCN2/CTGF mRNA is highly expressed in the embryoid body, then drops significantly in the blastocyst [89]. Expression then increases during the fetal stage, peaking in the neonate, and drops again in the infant, only to increase modestly throughout the juvenile and adult stages. Among all adult body sites, CCN2/CTGF is most highly expressed in the vasculature. In the healthy human eye, CCN2/CTGF is found in the vascular ECs of the iris, ciliary body, choroid, choriocapillaris, and the central retinal artery [90]. Using a transgenic GFP reporter mouse as a proxy for CCN2/CTGF expression, we have detected CCN2/CTGF:GFP signal at relatively high levels in the vasculature of the retina (in the superficial vascular plexus around the optic nerve head, and intermediate and deep capillary plexuses within the inner nuclear layer), choriocapillaris, the corneal endothelium and lens subcapsular epithelium [50]. ECs, mural cells (e.g., smooth muscle cells, pericytes), Müller cells, and retinal pigment epithelial cells (RPE) cells are major producers of CCN2/CTGF in retina of new born and adult mice while other cells such as astrocytes and microglia do not appear to be an important source of CCN2/CTGF [91]. CCN2/CTGF expression has been reported in activated astrocytes in the brain of human patients with Alzheimer’s disease suggesting a potentially pathogenic role in astrogliosis and neuroinflammation [92].

## 6. Constitutive and Inducible CCN2/CTGF Knockout Phenotype

Global knockout of CCN2/CTGF was found to be perinatal lethal. Ivkovic et al. showed that CCN2/CTGF^-/-^ mice died within minutes after birth as a result of skeletal defects and respiratory failure [93,94]. Other defects included impaired growth plate angiogenesis with formation of fewer intact capillaries, and disrupted organization of large blood vessels. Our recent studies revealed retinal hypovascularization and altered vascular permeability resulting from EC-specific loss of CCN2/CTGF [50]. CCN2/CTGF depletion perturbed genes and pathways that regulate proliferation, migration, and junction formation in ECs. Expression levels of IGF-1 TGF-β1, ΤGF-β2, FGF-10, and angiopoietin 1 were all diminished. Furthermore, several ECM proteins were downregulated, including laminin 4, SPARC, tenascin C, FACIT collagens, and proteoglycans. Many of these are critical for vascular BM integrity [50]. Thus, CCN2/CTGF signaling potentially contributes to the regulation of the vascular matrix composition and mechanical properties [27]. A study by Toda et al. has shown that partial reduction in CCN2/CTGF levels is well tolerated in adult mice with anti-glomerular BM glomerulonephritis [95]. These mice exhibited reduced proteinuria with ameliorated crescent formation and mesangial expansion suggesting that the regulation of CCN2/CTGF expression is important for disease manifestation.

## 7. CCN2/CTGF as a Mechanosensitive Gene

CCN2/CTGF was initially described as an immediate early gene expressed upon serum-stimulation of NIH3T3 cells [96]. Later, CCN2/CTGF was partially purified from the culture medium of human umbilical vein ECs (HUVECs) and named connective tissue growth factor due to its mitogenic and chemotactic activity on fibroblast-like cells in vitro [97]. At the time, CCN2/CTGF expression was found to be rapidly induced in response to TGF-β stimulation of fibroblasts [98]; thus, many early studies emphasized a potentially important role of CCN2/CTGF in the profibrotic and fibrogenic effects of TGF-β. The CCN2/CTGF gene has since been found to be transcriptionally regulated by various chemical and physical stimuli in different physio-pathological settings [99].

Of particular significance, the CCN2/CTGF gene was strongly up-regulated in mechanically challenged organs as a result of various etiologies (e.g., hypertension, hemodynamics, matrix stiffness, metabolic injury, and obstruction) [27,99,100,101]. The levels of CCN2/CTGF were increased several fold in atherosclerotic vessels experiencing altered hemodynamic forces compared to normal arteries [102]. CCN2/CTGF levels were also remarkably elevated in lung and vasculature in various experimental models of hypertension [103]. In addition, the specialized cases of abnormal scarring (e.g., keloids), which apparently develop in regions of the body that are subjected to relatively higher mechanical strains than others, were highly enriched in CCN2/CTGF [104]. The scar that persists in these lesions is itself a tissue under increased mechanical strain and contained abnormally high levels of CCN2/CTGF. In addition to mechanical stimuli, CCN2/CTGF gene expression was found to be upregulated by hypoxia and downregulated by nitric oxide [105]. Other metabolic factors such as hyperglycemia, reactive oxygen species, advanced glycation end products (AGEs), and free fatty acids induce CCN2/CTGF expression in affected tissues [106]. AGEs may induce CCN2/CTGF expression via the TGF-β pathway or via the receptor for AGEs-extracellular signal-regulated kinase (RAGE-ERK)/p38 mitogen-activated protein kinase-Smad cross-talk pathway [107]. Thus, mechanical factors typified by stiffness, tension, shear stress, stretch, and hydrostatic pressure and chemical stimuli such as hypoxia, hyperglycemia and hyperlipidemia might be the primary inducers of the CCN2/CTGF gene in vivo.

**Figure 4:**
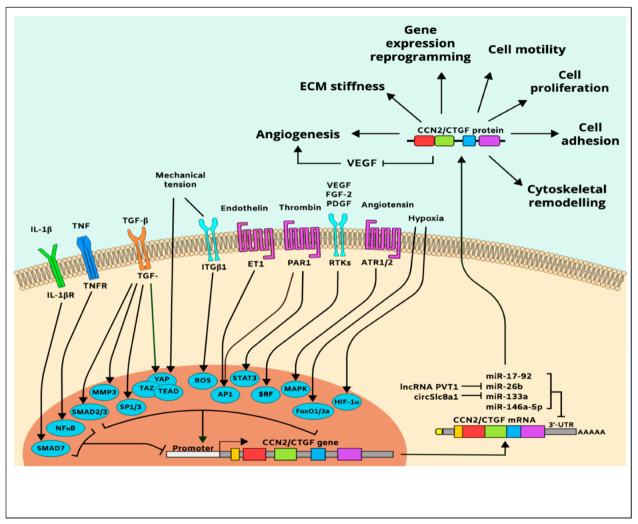
Simplified pathway map of CCN2/CTGF regulation and function.

In vitro cell culture studies further showed that the CCN2/CTGF gene is upregulated in responsive cells exposed to various natural molecules or synthetic compounds including VEGF, FGF-2, platelet-derived growth factor (PDGF) [107,108], angiotensin II, bone morphogenetic protein (BMP)-2, glucocorticoids, monocyte chemotactic protein-1 endothelin-1, secreted frizzled-like protein (sFRP)-2, antiproliferative factor, and thrombin [105,109]. Inflammatory cytokines, including tumor necrosis factor (TNF)-α and interferon (IFN)-γ, inhibit CCN2/CTGF gene expression [105], while histamine, serotonin, and prostaglandins [110] increase it. In addition, adenosine, α-tocopherol, nicotine, and harmine induce CCN2/CTGF expression, while curcumin and caffeine repress CCN2/CTGF gene expression indicating that the CCN2/CTGF gene is sensitive to various chemical and physical stressors [111]. A simplified map of CCN2/CTGF gene regulation and protein function is shown in Figure 4.

The CCN2/CTGF gene is predominantly regulated at the transcriptional level; its transactivation depends, on *PDZ*-binding motifs such as yes-associated protein (YAP)/transcriptional coactivator with PDZ-binding motif (TAZ)/transcriptional enhancer factor TEF-1 (TEAD) and ETS protooncogene 1 (Ets1) [112]. Other transcription factors that regulate CCN2/CTGF include hypoxia-inducible factor (HIF)-1α, matrix metalloproteinase (MMP)-3, activating protein (AP)-1, nuclear factor (NF)-κB, Smad, specificity protein (Sp)-1, serum response factor (SRF), forkhead box (FoxO3a), and signal transducer and activator of transcription (STAT) [105]. The effect of HIF-1α-induced CCN2/CTGF expression is particularly important because once CCN2/CTGF gene is induced under ischemic conditions, HIF1-α levels declined even though the hypoxic conditions were maintained [113]. This suggests that CCN2/CTGF is a downstream effector of hypoxia. Meanwhile, CCN2/CTGF is also a predicted and validated target of the miR-17-92 microRNA cluster that regulates tumor neovascularization, miR-146a-5p that suppresses angiogenesis and promotes joint stiffness [114], and the circular RNA circSlc8a1, which functions as an endogenous sponge for miR-133a, attenuating mechanical overload [115]. Additionally, the plasmacytoma variant translocation 1 (PVT1) long noncoding RNA promotes CCN2/CTGF expression and subsequent angiogenesis through binding to and degradation of miR-26b [116].

## 8. Mode of Action of the CCN2/CTGF Protein

The available evidence obtained from in vitro studies suggests that, CCN2/CTGF promotes various cell type- and context-dependent responses. In vitro studies showed that CCN2/CTGF binds multiple integrins (e.g., α_v_β_3_, α_5_β_1_) and non-integrin receptors including tropomyosin receptor kinase, Notch, lipoprotein receptor-related proteins (LRPs), HSPGs, the cation-independent mannose-6-phosphate receptor (M6P/IGF-2R), and tyrosine kinase (TK) receptors [107]. Many of the effects of CCN2/CTGF are achieved through the binding of integrin receptors, in some cases with LRPs and HSPGs acting as co-receptors [117]. CCN2/CTGF also binds ECM molecules (e.g., aggrecan, collagen, fibronectin, and fibulin), growth factors (e.g., VEGF, FGF, PDGF, and BMPs), and proteases (e.g., MMP-2, MMP-14, and kallikrein) [117,118,119,120]. The in vivo relevance of these interactions and their impact on the cellular and tissue responses are not known. CCN2/CTGF–integrin binding results in the activation of signaling cascades, including extracellular signal-regulated kinases (ERKs), phosphoinositide 3-kinase(PI3K), and small GTPases of the Rho family [107,121,122], which culminate in cytoskeletal actin remodeling, changes in cell shape and behavior and reprograming of gene expression. Interestingly, CCN2/CTGF expression is also dependent on activation of these signaling pathways [78,123,124], suggesting a regulatory feedforward loop in which CCN2/CTGF promotes the very cellular signaling processes that promote its expression and amplify its activity.

However, CCN2/CTGF occurs as either a full-length protein or truncated variants lacking different N- or C-terminal domains. The latter have been identified in tissue cultures or body fluids, and these fragments may retain biological activities either similar to or distinct from the intact protein. A 10-kDa form of CCN2/CTGF that includes the cysteine-knot motif has been shown to elicit a full-length CCN2/CTGF protein-like activity [125]. Mokalled et al. have shown that overexpression of a CCN2/CTGF truncated form containing the C-terminal TSP1 and CT domains only was as effective as the full-length protein in mediating spinal cord regeneration in zebra fish [126]. On the other hand, truncated variants of CCN2/CTGF containing IGFBP and vWC domains were found in the vitreous of patients with active proliferative diabetic retinopathy [127,128]. Furthermore, a study by Kaasbøll et al. suggested that CCN2/CTGF could be synthesized and secreted as a preproprotein that is autoinhibited by its two N-terminal domains (IGFBP and vWC) and the subsequent proteolytic processing and homodimerization of the TSP1 and CT domains produce a fully biologically active form of CCN2/CTGF [129]. Taken together, these findings suggest that the multimodular organization of the CCN2/CTGF protein is a means of generating through proteolytic cleavage, CCN2/CTGF variants, which may either recapitulate a loss of function of the full-length protein or exhibit a diverse range of biological activities in a tissue- and context-dependent manner.

## 9. Role of CCN2/CTGF in Eye Diseases

### 9.1. DR

DR has often been described as a purely vascular disorder of the retina where VEGF is the key factor driving microvascular damages from non-proliferative to proliferative DR [130]. Numerous studies described differential expression of CCN2/CTGF gene in response to hyperglycemia and suggested a potentially important role of CCN2/CTGF in the pathogenesis of diabetes, from the initial metabolic insult(s) (e.g., hyperglycemia, obesity, insulin resistance and deficiency), to end-organ complications/failure [131,132]. Because CCN2/CTGF plays a major role in maintaining the ECM and wound healing, a greater emphasis has been placed on its involvement in diabetes-induced ECM remodeling [69,133]. Thickening of the retinal capillary basal lamina (BL), a part of the BM, is a hallmark of the pre-clinical phase of DR, and multiple studies have suggested a role of CCN2/CTGF in BL thickening, which has been attributed to increased formation of AGEs, most likely via TGF-β-induced CCN2/CTGF expression [131,134]. In CCN2/CTGF^+/−^ mice, reduced CCN2/CTGF protein levels prevented BL thickening of retinal capillaries in streptozotocin (STZ)-induced diabetes [135]. CCN2/CTGF^+/−^ mice showed no BL thickening, reduced pericyte loss, and formation of acellular capillaries 8 months after diabetes induction despite good glycemic control, suggesting a regulatory role of CCN2/CTGF in ECM remodeling at the early stages of diabetes [134]. However, considerable retinal neuronal damage precedes microvascular alterations in the early stages of DR indicating that DR may also be viewed as a neurodegenerative disease of the retina [136]. Retinal neurons are the most vulnerable and metabolically demanding cells in the retina and are the first to be affected by changes in the microenvironment. Interestingly, a study by Tikellis et al. indicated that, in diabetic rats, CCN2/CTGF was localized mainly to the ganglion cell layer, where its expression was nearly two-fold that of non-diabetic controls, suggesting that CCN2/CTGF plays a pivotal role in mediating diabetes-associated retinal neuronal pathology [137]. Our own preliminary studies have identified CCN2/CTGF as an important component of the neuronal progenitor cells in the retina and other studies have localized CCN2/CTGF in neurons and glial cells in the brain [138]. One mechanism of DR-associated CCN2/CTGF induction in neuronal cells was investigated in Period 2 (Per2)-mutant mice, which recapitulate a DR-like retinal vascular phenotype [139]. In Per2 mutants, β-catenin localized to the nucleus and transactivated the CCN2/CTGF gene. However, the functional significance of CCN2/CTGF expression in neuronal cells remains to be determined.

CCN2/CTGF has also been portrayed as a fibrotic factor and a high CCN2/VEGF ratio in eyes with proliferative DR tips the balance from neovascularization to fibrotic membrane formation, ultimately leading to fibrovascular membrane contraction and tractional retinal detachment [140]. While the role of CCN2/CTGF in the regulation of BM protein turnover is well evidenced in vascular and non-vascular pathologies [140], its profibrotic effects are more nuanced. Transgenic models of CCN2/CTGF overproduction in different tissues exhibited various phenotypes including no fibrotic reaction, mild fibrosis or clear fibrotic phenotypes depending on the expression site/organ and levels of CCN2/CTGF [141]. Complicating the matter is a finding by our group and others that the CCN proteins including CCN2/CTGF are highly susceptible to proteolytic degradation by various proteases and collagenases [74,142]. Sure enough, a study by Hinton et al. showed that only an N-terminal fragment of the CCN2/CTGF molecule was stably expressed in proliferative DR [127,143]. Although the in vivo biological relevance and activities, if any, of such a truncated variant remains to be determined, disease models of retinopathies seem to recapitulate a loss of function phenotype of the full CCN2/CTGF protein. This may undermine a pathogenic function of CCN2/CTGF in inflammatory diseases such as proliferative DR.

### 9.2. Proliferative Vitreoretinopathy

Proliferative vitreoretinopathy (PVR) is characterized by the formation of contractile membranes within the vitreous and along the preretinal and subretinal surfaces [144]. This retinal scarring impairs neural transmission and increases the likelihood of detachment and loss of vision. Indeed, following retinal detachment surgery, a fibrous membrane may grow on the subretinal or epiretinal surface of the eye, causing the retina to detach from the posterior pole of the eye. This PVR is the major cause of failure following retinal detachment surgery, with an estimated incidence of 5–10% [145]. PVR membranes grow in a four-stage process. Vitreous haze and pigment clumps characterize the first stage. In the second stage, the retina stiffens, wrinkles, and shows breaks with rolled edges. Retinal vessels become tortuous. In the third stage, full-thickness retinal folds involve 1-3 quadrants. Finally, the fourth stage is marked by retinal folds involving all four quadrants and retinal detachment. PVR membranes are composed of multiple cell types, including RPE, fibroblasts, glial cells, and macrophages, though RPE and glial cells are the most prominent and are thus presumed to contribute significantly to membrane development [146]. Several growth factors have been identified in PVR membranes, including CCN2/CTGF, hepatocyte growth factor (HGF), and PDGF [147]. Among these, CCN2/CTGF is the most influential, as it is present during all stages and is dramatically upregulated in glial cells in late stage PVR [146]. In fact, CCN2/CTGF accumulates in the subretinal fluid of retinal detachment patients even before the onset of PVR. CCN2/CTGF is induced following RPE injury in vitro and increases the migratory potential of RPE cells in a calcium-dependent manner [148]. However, much remains to be learned about the precise role of CCN2/CTGF and its truncated variants in the pathogenesis of PVR and the mechanistic link between CCN2/CTGF signals retinal gliosis and fibrosis [149].

### 9.3. AMD and Choroidal Neovascularization

Neovascular or exudative AMD is the leading cause of vision loss in the elderly [150]. The wet form of AMD occurs as a result of abnormal blood vessel growth in the choriocapillaris (i.e., choroidal neovascularization or CNV), ultimately leading to fluid leakage, exudates, and/or hemorrhages below the macula, a region near the center of the retina with specialized cells for high-acuity vision [151]. Development of CNV is commonly associated with a disruption of Bruch’s membrane as a result of a traumatic break, degeneration of the RPE, tissue traction, and/or inflammation [152]. Choriocapillary ECs, pericytes, fibroblasts, and inflammatory cells invade the subretinal space causing abnormal choroidal neovessel growth and tissue remodeling/scarring that culminates in the formation of a choroidal neovascular membrane. Subsequently, the metabolism of the RPE layer changes. This results in protein, lipid, and AGE accumulation above Bruch’s membrane. Accumulated proteins may deposit over Bruch’s membrane as drusen, exacerbating the environmental dysregulation. RPE cells transition to myofibroblasts or mesenchymal-like cells that construct epiretinal membranes within a transient ECM. These membranes exert upward tensional forces on the attached underlying retina, leading to retinal detachment and ultimately blindness.

High CCN2/CTGF levels were found in surgically excised human choroidal neovascular membranes, especially within choroidal ECs and RPE cells [153]. Concordantly, inhibition of CCN2/CTGF resulted in a significant reduction in the choroidal neovascular membrane associated with sub-retinal fibrosis [154] suggesting a pathogenic role of CCN2/CTGF in the development of fibrovascular subretinal membranes. CCN2 promoted changes in RPE behavior and function including their differentiation state, proliferation, migration, matrix synthesis, enzyme production, and contraction [155]. Ramos de Carvalho et al. found a link between CCN2/CTGF signals, proteasome activity and pro-fibrogenic mechanisms in the RPE, which could suggest the therapeutic potential of proteasome-modulating agents in RPE-mediated fibrotic responses [156]. Interestingly, non-selective inhibition of CCN2/CTGF gene expression through cytoskeletal cell disruption reduced CNV lesion size and fibrosis by up to 60% in a mouse model of CNV [157]. Clearly, CCN2/CTGF may hold a particular position at the crossroad of neoangiogenesis and fibrosis associated with CNV.

### 9.4. Glaucoma

Glaucoma, the second leading cause of irreversible blindness worldwide, is an optic neuropathy characterized by optic disk damage and visual field loss resulting from death of RGCs in the inner retina and loss of their axons in the optic nerve [158]. Open-angle glaucoma is a chronic disease that may be clinically silent for years until irreversible damage has taken place. In primary open-angle glaucoma (POAG), impaired drainage of aqueous humor through the trabecular meshwork (TM) and Schlemm’s canal results in elevated intraocular pressure (IOP), the greatest risk factor for disease development. This generates stress in the sclera and optic nerve head and disrupts blood flow, leading to damage of the optic nerve. While the mechanisms are not completely understood, there is some evidence that changes in the trabecular meshwork ECM and actomyosin contribute to impaired drainage of the aqueous humor.

CCN2/CTGF is constitutively expressed by cells of the TM and its levels were reported to significantly increase in the aqueous humor of glaucoma patients compared to control individuals [159]. The lens-specific overexpression of CCN2/CTGF in mice led to an increase in the IOP, which was further accompanied by reactive gliosis and functional loss of RGCs, inner nuclear cells, and photoreceptors [159] Functionally, the effects of CCN2/CTGF have been largely attributed to its profibrotic effects acting, at least in part, as a downstream effector of TGF-β [160]. It was suggested that TGF-β/CCN2 signaling culminates in the differentiation of juxtacanalicular mesenchymal cells into myofibroblasts, which produce excessive amounts of ECM proteins causing the TM tissue to stiffen and the outflow facility to decrease [161]. However, as shown in our studies, CCN2/CTGF signals affect the expression of diverse genes and pathways including cytoskeletal and ECM proteins, growth factors, and transcriptional co-regulators [50]. Therefore, it remains unclear whether CCN2/CTGF overexpression is an adaptive response to increased mechanical stress (i.e., IOP) on TM cells or causative of ECM stiffening and subsequent IOP increase. In human TM cells subjected to heat shock or oxidative stress, CCN2/CTGF were elevated and this both increased contractility of the trabecular meshwork and improved cell viability [162]. Further rheological studies are needed to tease apart the deleterious and protective effects of CCN2/CTGF on the stressed TM.

### 9.5. Retinitis Pigmentosa

In a mouse model of retinitis pigmentosa, photoreceptor degeneration coincides with reactive gliosis in Müller cells, which triggers Müller cell activation of the Hippo pathway components YAP/TEAD and subsequent upregulation of CCN2/CTGF [163]. In species with regenerative properties, like zebrafish, Müller cells may, under certain conditions, dedifferentiate to retinal stem-like cells that can subsequently proliferate to generate neurons [164]. Although this process is not effective in mammals, it is possible that Müller cell upregulation of CCN2/CTGF is an attempt to assuage damage to the neural retina. On the other hand, prolonged reactive gliosis can be detrimental. Thus, it remains to be seen whether the role of CCN2/CTGF is pathological or regenerative in the context of photoreceptor loss.

### 9.6. Corneal Diseases

The cornea is a transparent avascular tissue made of an epithelium and Bowman’s membrane anteriorly, and an endothelium with its basement membrane, Descemet’s membrane posteriorly, sandwiching a stroma. These components play an important role in maintaining corneal clarity, structure, and function. The most common corneal diseases involve corneal scarring following acute injury or vision correction surgery which can lead to vision degrading opacification of the cornea [165]. Moreover, human corneal ECs, which are unable to regenerate in vivo, are lost due to aging, trauma or endothelial disorders such as Fuchs endothelial corneal dystrophy (FECD) [113]. Consequently, surviving ECs enlarge and slide along the DM to cover the areas of lost corneal ECs. Such a dysfunctional endothelium causes corneal edema and vison loss. Therapeutic approaches to address these pathological conditions are the subject of intense investigations.

CCN2/CTGF expression was detected in the basal layers of the epithelium, stromal keratinocytes, and corneal ECs [166]. In mice, wounded corneas responded with an immediate upregulation of CCN2/CTGF in the epithelium at the wound margin and a sustained CCN2/CTGF induction during re-epithelialization [167]. Inducible loss of CCN2/CTGF function delayed but did not prevent epithelial healing. The precise function of CCN2/CTGF in this process remains to be elucidated. Similarly, the regulation and function of CCN2/CTGF in FECD, which can be caused by diverse genetic defects that produce a virtually identical clinical phenotype, is unknown [168]. Aberrant deposition of ECM proteins occurs in FECD, manifested as thickening of the DM, but the potential roles of CCN2/CTGF in these alterations are yet to be investigated. Like the CCN2/CTGF gene, several genes implicated in the pathogenesis of FECD are known to induce epithelial mesenchymal differentiation [169]. Further studies are needed to elucidate the precise role of CCN2/CTGF in the pathogenesis of FECD and its usefulness as a therapeutic target to treat and/or prevent corneal conditions.

## 10. Conclusions

The data discussed in this paper clearly indicate that the vascular matrix is a determining factor of vascular growth, morphogenesis and barrier function during blood vessel development and regeneration and that qualitative and/or quantitative alterations of components of the vascular matrisome have dramatic pathophysiological consequences on retinal health and function. The major ECM components of the BM are well recognized to control vascular tissue organizational features and physical and chemical properties. However, it is also becoming clear that although subendothelial matrix components, particularly those of the matricellular protein family (e.g., CCN2/CTGF), do not subserve a structural role in the vascular wall, they are critical for proper tissue vascularization and cellular adaptational responses to chemical and physical challenges. Chemical, mechanical, and proteolytic insults associated with diabetes, ischemic injury and/or inflammation compromise the regulation and function of the matricellular protein CCN2/CTGF leading to consequential changes in the physical properties of the vascular wall in a myriad of retinal microvascular diseases. CCN2/CTGF has been suggested to be a fulcrum over which hangs a balance between neoangiogenesis and fibrosis. However, the mechanisms whereby CCN2/CTGF regulates both vascular function and dysfunction in vivo are not well understood. CCN2/CTGF is functionally diverse eliciting specific activities in a context-specific manner. CCN2/CTGF signals are likely critical for the expression and maintenance of a dynamically evolving vascular matrix but also for the regulation of the flow of information between cells and the extracellular milieu. As such, CCN2/CTGF may act as a key factor allowing cells to maintain nearly constant structural and mechanical properties in the face of growth, differentiation and ECM turnover. The CCN2/CTGF signals depend largely on its interactome which includes ECM proteins, integrins and growth factor receptors. Vascular tissue function and integrity may depend not only on the availability of CCN2/CTGF interacting partners, but also on the physical state (i.e., intactness) of the CCN2/CTGF protein itself. Since CCN2/CTGF is highly susceptible to proteolytic degradation that commonly occurs during inflammatory reactions and vasculopathies, the manifestation of these diseases may actually recapitulate the tissue reaction to the loss of specific CCN2/CTGF signals. Therefore, the question of whether CCN2/CTGF is a “friend” or “foe” in microangiopathies is still outstanding. Moreover, our understanding of the vascular and non-vascular matrix in the retina is limited and much remains to be learned about how key ECM proteins like those of the CCN family contribute to the short- and long-term robustness and optimal function of the retina as a whole. Such building blocks of knowledge would likely help uncover new targets and develop new tools for controlling the CNS physical and chemical environment in a therapeutic context.
